# Genome Sequencing of SAV3 Reveals Repeated Seeding Events of Viral Strains in Norwegian Aquaculture

**DOI:** 10.3389/fmicb.2020.00740

**Published:** 2020-04-24

**Authors:** Michael D. Gallagher, Marius Karlsen, Elin Petterson, Øyvind Haugland, Iveta Matejusova, Daniel J. Macqueen

**Affiliations:** ^1^School of Biological Sciences, University of Aberdeen, Aberdeen, United Kingdom; ^2^The Roslin Institute and Royal (Dick) School of Veterinary Studies, The University of Edinburgh, Edinburgh, United Kingdom; ^3^PHARMAQ AS, Oslo, Norway; ^4^Marine Laboratory, Marine Scotland Science, Aberdeen, United Kingdom

**Keywords:** salmonid alphavirus, aquaculture, phylogeography, molecular epidemiology, evolution

## Abstract

Understanding the dynamics of pathogen transfer in aquaculture systems is essential to manage and mitigate disease outbreaks. The goal of this study was to understand recent transmission dynamics of salmonid alphavirus (SAV) in Norway. SAV causes significant economic impacts on farmed salmonids in European aquaculture. SAV is classified into six subtypes, with Norway having ongoing epidemics of SAV subtypes 2 and 3. These two viral subtypes are present in largely distinct geographic regions of Norway, with SAV2 present in Trondelag, SAV3 in Rogaland, Sogn og Fjordane, and Hordaland, and Møre og Romsdal having outbreaks of both subtypes. To determine likely transmission routes of Norwegian SAV an established Nanopore amplicon sequencing approach was used in the current study. After confirming the accuracy of this approach for distinguishing subtype level co-infections of SAV2 and SAV3, a hypothetical possibility in regions of neighboring epidemics, twenty-four SAV3 genomes were sequenced to characterize the current genetic diversity of SAV3 in Norwegian aquaculture. Sequencing was performed on naturally infected heart tissues originating from a range of geographic locations sampled between 2016 and 2019. Phylogenetic analyses revealed that the currently active SAV3 strains sampled comprise several distinct lineages sharing an ancestor that existed ∼15 years ago (95% HPD, 12.51–17.7 years) and likely in Hordaland. At least five of these lineages have not shared a common ancestor for 7.85 years (95% HPD, 5.39–10.96 years) or more. Furthermore, the ancestor of the strains that were sampled outside of Hordaland (Sogn of Fjordane and Rogaland) existed less than 8 years ago, indicating a lack of long-term viral reservoirs in these counties. This evident lack of geographically distinct subclades is compatible with a source-sink transmission dynamic explaining the long-term movements of SAV around Norway. Such anthropogenic transport of the virus indicates that at least for sink counties, biosecurity strategies might be effective in mitigating the ongoing SAV epidemic. Finally, genomic analyses of SAV sequences were performed, offering novel insights into the prevalence of SAV genomes containing defective deletions. Overall, this study improves our understanding of the recent transmission dynamics and biology of the SAV epidemic affecting Norwegian aquaculture.

## Introduction

Salmon pancreas disease virus (SPDV), commonly known as salmonid alphavirus (SAV) is a major economically damaging pathogen of European salmonid aquaculture, causing pancreas disease (PD) in Atlantic salmon, and sleeping disease (SD) in freshwater rainbow trout (Weston et al., 1999; McLoughlin and Graham, 2007). SAV is a (+)ssRNA virus (family *Togaviridae*) with a ∼12 kb genome consisting of two open reading frames (ORFs), encoding the structural polyprotein (∼4 kb), and the non-structural polyprotein (∼8 kb) (Weston et al., 2002). The viral genome exists as a polyadenylated ∼12 kb genomic RNA molecule from which the non-structural polyprotein is translated, and a transcribed ∼4 kb sub-genomic RNA, also polyadenylated, from which the structural polyprotein is translated (Weston et al., 1999, 2002; Villoing et al., 2000).

Six subtypes of SAV have been identified by phylogenetic analysis (SAV1-6) (Fringuelli et al., 2008; Graham et al., 2012), which are to some extent separated geographically, with Scotland reporting cases of SAV1, SAV2, SAV4, and SAV5, Ireland reporting cases of SAV1, SAV2, SAV5, SAV6 (Graham et al., 2012), and Norway presenting sustained epidemics of SAV2 and SAV3 (Hodneland et al., 2005; Hjortaas et al., 2013). Previous work has shown that the different subtypes diverged prior to the onset of modern aquaculture, and that it is likely that each SAV subtype had independent introductions to farmed salmonids (Karlsen et al., 2014). Additionally, multiple wild fish species testing positive for SAV have been identified as being potential viral reservoirs including common dab (*Limanda limanda*), long rough dab (*Hippoglossoides platessoides*), European plaice (*Pleuronectes platessa*), and ballan wrasse (*Labrus bergylta*) (Snow et al., 2010; Bruno et al., 2014; McCleary et al., 2014; Ruane et al., 2018), with the ancestral source of SAV likely being centered in the North Sea (Karlsen et al., 2014). Such discoveries have shown that salmonids are not the exclusive host range of SAV, but is instead present in a range of other fish species, though SAV has not yet been shown to cause mortalities in non-salmonids.

Similar to other RNA viruses, the high rate of evolution in SAV can be detected at a genetic level within timeframes of a few years (Karlsen et al., 2014, 2006). Previous work on Norwegian SAV3 showed the presence of two co-circulating strains that overlap both temporally and spatially, across wide geographic distances, indicating significant genetic diversity within SAV3 available for molecular epidemiological studies (Karlsen et al., 2014). Both SAV subtypes present in Norway are geographically structured with the counties of Rogaland and Hordaland having a SAV3 epidemic, Trøndelag having a SAV2 epidemic, and Møre og Romsdal reporting cases of both SAV2 and SAV3 (Norwegian Veterinary Institute, 2018). While the overwhelming majority of SAV cases in Sogn og Fjordane are SAV3, a small number of SAV2 outbreaks have occurred (Norwegian Veterinary Institute, 2018). These regions of overlapping epidemics provide ample opportunity for co-infections to arise between the two subtypes, observed recently in Møre og Romsdal in a case of PD (Norwegian Veterinary Institute, 2018).

Both the transport of fish stock and passive drift along water currents are suggested to be involved in the spread of SAV strains (Karlsen et al., 2006; Kristoffersen et al., 2009; Viljugrein et al., 2010). However, it is likely that the two routes of viral transmission play roles at different spatial and temporal scales. While passive drift along water currents is probably important in local outbreak clusters and for several years at a time, anthropogenic transport of virus (i.e., the movement of fish stock) may play a more important role in viral transmission across large geographic distances and the seeding of previously uninfected regions. Assuming the major explanatory factor for SAV3 transmission was transport by water currents, we would expect to recover geographically distinct groups of SAV in a phylogenetic analysis, particularly when considering the high evolutionary rate of SAV (Karlsen et al., 2014) and length of the SAV3 epidemic in Norway (Poppe et al., 1989; Hjortaas et al., 2016). However, it has not yet been possible to test alternative predictions about SAV transmission routes, as the most recent publicly available genome sequences from Norwegian SAV3 were sampled in 2010 (Petterson et al., 2013), leaving the current status of SAV genetic diversity and SAV evolutionary dynamics in Norwegian aquaculture poorly characterized.

Therefore, to characterize the current phylogenetic structure of SAV3 and thus test the hypothesis that passive drift by water currents has been the dominating transmission mechanism, an established Nanopore amplicon sequencing approach (Gallagher et al., 2018) was used in the current study. After first confirming the accuracy of this approach for distinguishing sub-type level co-infections of SAV2 and SAV3, which we considered a hypothetical possibility in regions with overlapping epidemics, twenty-four SAV3 genomes were sequenced from heart tissues sampled between 2016 and 2019. This dataset allowed us to characterize the current genetic diversity of SAV3 in Norwegian aquaculture. We found that several distinct lineages of SAV3, representing independent transmission chains, are active in Norway. Lack of clear geographical structuring in these lineages further suggested that anthropogenic transport of the virus likely played a significant role in shaping the current genetic structure and is an important transmission route that acts not only over large geographic distances, but also locally.

## Materials and Methods

### Sample Preparation and PCR

Twenty-three naturally SAV-infected heart tissues of Atlantic salmon and one heart tissue from rainbow trout stored in RNAlater were obtained from PHARMAQ Analytiq (Zoetis) (Table 1). Samples were selected from across as wide a geographic range as possible and were sampled between 2016 and 2019. Total RNA was extracted from each sample using a phenol-chloroform approach, and integrity assessed using gel electrophoresis. cDNA was synthesized using Protoscript II Reverse Transcriptase (New England Biolabs) and a mix of random hexamer and anchored-dT (dT_23_VN) primers. First strand cDNA was used as template for PCR reactions.

**TABLE 1:** Details of the SAV-infected tissue samples used in this study.

**Sample ID**	**Accession**	**Location**	**Day**	**Month**	**Year**	**Species**	**Average Coverage**
FR14696209	MN906920	Rogaland	8	3	2018	A. salmon	5,730
FR12429232	MN906917	Hordaland	20	2	2017	A. salmon	12,434
FR12428008	MN906916	Hordaland	30	1	2017	A. salmon	9,668
FR13804133	MN906918	Sogn of Fjordane	16	1	2017	A. salmon	12,331
FR14304869	MN906919	Sogn of Fjordane	13	12	2017	A. salmon	9,491
FR10484738	MN906915	Sogn of Fjordane	14	6	2016	A. salmon	8,483
FR14308528	MN906921	Hordaland	2	1	2018	A. salmon	8,073
FR14305164	MN906922	Hordaland	24	1	2018	A. salmon	4,713
FR16893400	MN906923	Hordaland	13	3	2018	A. salmon	6,510
FR16893417	MN906924	Hordaland	13	3	2018	A. salmon	10,273
FR16893355	MN906925	Hordaland	13	3	2018	R. trout	5,184
FR14700631	MN906926	Sogn og Fjordane	22	5	2018	A. salmon	4,783
FR14700646	MN906927	Sogn og Fjordane	22	5	2018	A. salmon	7,911
FR14603719	MN906928	Hordaland	7	6	2018	A. salmon	5,340
FR14695472	MN906929	Sogn og Fjordane	31	8	2018	A. salmon	7,314
FR16934408	MN906930	Hordaland	27	8	2018	A. salmon	2,829
FR16934406	MN906931	Hordaland	27	8	2018	A. salmon	4,506
FR18260741	MN906932	Hordaland	13	2	2019	A. salmon	6,053
FR18260737	MN906933	Hordaland	13	2	2019	A. salmon	3,455
FR18290543	MN906934	Sogn og Fjordane	15	4	2019	A. salmon	1,089
FR16900370	MN906935	Sogn og Fjordane	29	4	2019	A. salmon	7,555
FR18295490	MN906936	Hordaland	30	4	2019	A. salmon	5,396
FR14794986	MN906937	Hordaland	31	1	2018	A. salmon	1,561
FR18295483	MN906938	Hordaland	30	4	2019	A. salmon	4,323

*Average coverage represents sequencing depth across genome.*

Viral genomes were amplified in six overlapping PCR amplicons of roughly 2 kb in length (primer sequences available in Table 2) using LongAmp polymerase (New England Biolabs) with the following PCR cycling conditions: 30 s at 94°C, followed by 35 cycles of 15 s at 94°C, 1 min at 56°C and 2 min 15 s at 65°C, with a final extension for 10 min at 65°C. Primers were designed in regions of the SAV genome conserved between SAV2 and SAV3 (Table 2). All PCR products were visualized on a 1% agarose gel before being excised and purified using Monarch Gel Extraction Kit (New England Biolabs), eluted in 10 μl of elution buffer and stored at −80°C until sequencing.

**TABLE 2:** Details of PCR primers used to amplify SAV in overlapping amplicons.

**Primer name**	**Primer sequence 5′ – 3′**	**Amplicon length**
Amplicon 1	AGACTGCGTTTCCAGGGTTC	2156
	CCCGTAGATGCCAATCGTGT	
Amplicon 2	GAATACGTTTACGAATTGTCCTCC	1966
	ACCGAGACGGACTTGAAATACC	
Amplicon 3	GACCTGGTGTTTTGTGACGC	2438
	TCCCGTGTTAGCCCTCTAGG	
Amplicon 4	GCAGCGTCCACRGCCATAGT	2014
	CATCAGGCGTTTTACAGGGTC	
Amplicon 5	TTGTGGCGGCTTCCTGTTAC	2110
	GTAAACGTCTGGGAGTCGCTG	
Amplicon 6	AGAGAACGCAGCAAGGGC	2402
	GGCACTTCTTCACCACGCA	

### MinION Library Preparation

Five hundred ng of PCR product from each sample, quantified with the Qubit dsDNA HS Assay Kit (Thermo Fisher Scientific), was used as the input for MinION library preparation with the Ligation Sequencing Kit 1D SQK-LSK109. DNA was end-repaired with NEBNext Ultra II End Repair/dA Tailing kit (New England Biolabs) and purified with AMPure XP beads (Beckman Coulter) in a 1:1 ratio before being eluted in 25 μl of nuclease-free water (Sigma-Aldrich). 350 ng of each cleaned DNA sample was barcoded using the Native Barcoding Expansion 1-12 and 13-24 kits (ONT: EXP-NBD104 and EXP-NBD114) and Blunt/TA Ligation Master Mix (New England Biolabs) before being purified with AMPure XP beads in a 1:1 ratio and eluted in 26 μl of nuclease-free water. The barcoded samples were pooled in equal quantities to a total of 250 ng in 45 μl of water. Sequencing adapters (AMII – ONT) were ligated to the DNA using Blunt/TA Ligation Master Mix before being purified with AMPure XP beads in a 1:2 ratio and washed with Short Fragment Buffer (SFP) (ONT) before being eluted in 15 μl of elution buffer. Samples were sequenced in two libraries on separate R9.4.1 MinION flow cells (ONT) (Table 1) without live basecalling.

### Data Analysis

MinION sequence basecalling and demultiplexing was performed with Guppy v3.1.5 using the high accuracy basecaller on a Linux CPU system with default parameters. Resulting FASTQ files were aligned to reference sequences for SAV2 (MH708652) and SAV3 (JQ799139) simultaneously using MiniMap2 (Li, 2018) with default parameters. Resulting alignment files were visualized in Geneious v.2019.0.4 and consensus sequences were generated using the “Highest Quality” threshold parameter. Consensus sequences were inspected manually for alignment errors or frameshift mutations that would disrupt the protein coding sequence of the genome.

FASTQ files were also aligned to a reference genome using the NGMLR mapper and analyzed for structural variants (deletions, duplications, and inversions) using Sniffles (Sedlazeck et al., 2018) with the following parameters: a minimum coverage of 50 reads per variant and a minimum variant size of 10 bp. Anything smaller than 10 bp was not reliably detected by this software due to the prevalence of random indels generated during Nanopore sequencing. Resulting variant calling files were visualized in IGV and structural variants were manually inspected to reduce false positive calls.

Phylogenetic analysis was performed using the twenty-four consensus sequences generated in this study as well as all previously published whole genome sequences of SAV3 strains with sampling dates and locations, obtained from NCBI (Table 3). Sequences were aligned using MAFFT v.7 with default parameters (Katoh and Standley, 2013) and the best fit substitution model was determined using IQTREE (Nguyen et al., 2015; Kalyaanamoorthy et al., 2017) (TIM2 + G4; Posada, 2003). To further imply the evolutionary rate and divergence estimations, a regression of root-to-tip genetic distances against date of sampling was performed using TempEst (Rambaut et al., 2016) with the input tree generated in IQTREE (Nguyen et al., 2015) with the same best fit substitution model as above. A 11,681 bp alignment of 37 sequences was used for phylogenetic analysis in BEAST2 (Bouckaert et al., 2014) using tip-date time calibration, an uncorrelated relaxed clock model (Drummond et al., 2006), and a Coalescent Bayesian Skyline tree prior (Drummond et al., 2005). Ancestral state location reconstruction was used to estimate the likely geographic location of each node of the tree (Lemey et al., 2009). A single MCMC chain was run for 200 million generations, sampled every 20,000 generations and sampling convergence was confirmed with Tracer v1.7.1, evidenced by effective sample sizes >200 for all parameters (Rambaut et al., 2018). A maximum clade credibility tree was created using TreeAnnotator v2.5.1 (Drummond et al., 2012) after removing the first 10% of trees as burn-in. The resulting trees were visualized in FigTree v1.4.4.^1^

**TABLE 3:** Additional SAV3 genome sequences used in phylogenetic analysis.

**Isolate**	**Location**	**Sampling date**
AY604235	Hordaland	2003
AY604236	Hordaland	2002
AY604237	Hordaland	1997
AY604238	Sogn og Fjordane	2003
KC122918	Rogaland	2009
KC122919	Rogaland	2010
KC122920	Hordaland	2010
KC122921	Rogaland	2010
KC122922	Hordaland	2010
KC122923	Sogn og Fjordane	2010
KC122924	Troms	2010
KC122925	Møre og Romsdal	2010
KC122926	Møre og Romsdal	2010

### Validation of Nanopore Sequencing to Detect Subtype-Level Co-infections

As several of the samples sequenced in this study were from regions where both SAV2 and SAV3 have been detected, the effectiveness of this sequencing method at detecting subtype-level co-infections was determined. This rationale also follows our recent work, which provided evidence for SAV subtype-level infections in the same samples using a short-read sequencing approach (Gallagher et al., 2020). In the current study, Nanopore reads from samples of confirmed single-subtype infections were individually mapped to a structural polyprotein reference sequence of the relevant SAV subtype using MiniMap2 (Li, 2018) with default parameters. Mapped reads were extracted and only reads of >1,500 bp were used for subsequent analyses. Reads from each sample were sequenced on separate flow cells and so were labeled with a different run ID. To simulate subtype-level co-infections, reads were combined in different proportions from each sample so that each “co-infection” had 10,000 reads in total ranging from 5% SAV2 reads to 95% SAV2 reads, and the corresponding ratio of SAV3 reads. These artificial “co-infections” were then simultaneously mapped to reference sequences of both SAV2 and SAV3 using MiniMap2 and default parameters. The alignment files were visualized in Geneious v.2019.0.4 and the number of reads that mapped to the incorrect reference was calculated as the mapping error rate.

## Results

We sequenced 24 SAV3-infected fish hearts from Norway using an overlapping PCR amplicon approach and the MinION Nanopore platform (Table 1). This resulted in an average coverage of 6,459x across samples (minimum average coverage of 1,089x and maximum average coverage of 12,434x); significantly more than the minimum requirement for high consensus sequence accuracy (Gallagher et al., 2018). Of the 24 samples sequenced, near full genomes were recovered from 21 samples (approximately 11,600 bp in length), while partial genomes were recovered from 3 samples (∼ 9,500 bp). Overall, the SAV sequences generated in this study were found to be highly conserved, with an average nucleotide and amino acid similarity of the SAV3 sequences being 99.7% and 99.8%, respectively.

### Validation of Nanopore Sequencing to Detect Subtype-Level Co-infections

Combining Nanopore reads from single-subtype infections sequenced using the above approach allowed us to test this method’s ability to correctly detect SAV subtype level co-infections by mapping reads simultaneously to multiple reference genomes. A similar method has been used recently to provide evidence of SAV co-infections using high accuracy Illumina data (Gallagher et al., 2020), but its applicability to error-prone long-reads was not previously established. Reads from SAV2 and SAV3 were combined in a range of ratios, producing bioinformatic mimics of co-infection scenarios. All ratios of SAV2:SAV3 tested resulted in highly accurate mapping with less than 0.3% of the reads being mapped to the incorrect reference sequence across all samples (mean: 0.16% error rate). All naturally infected samples sequenced in this study were analyzed with this approach to detect any subtype co-infections, however, all samples proved to be single subtype in origin.

### Evolutionary Rate Analysis

Heterochronous gene sequences (i.e., sequences sampled at different time points) can be used to infer time-constrained phylogenies, especially those of rapidly evolving RNA viruses. However, for reliable estimation of a time-scaled phylogenetic tree, sequences should contain enough temporal signal to reconstruct the relationship between time and genetic distance (Rambaut et al., 2016). To determine whether such an analysis is appropriate for this data, and to estimate the evolutionary rate of SAV3, an analysis on the clock-like behavior of SAV was performed using TempEst. A root-to-tip regression analysis showed that the whole dataset (37 SAV3 genome sequences; 11,861 bp alignment) showed temporal signal (correlation coefficient, 0.765) and was subsequently used to estimate the evolutionary rate of SAV3. The evolutionary history was reconstructed with a relaxed molecular clock and a coalescent skyline population demographic model (Drummond et al., 2005). The estimated evolutionary rate for SAV3 was 7.351 × 10^–5^ substitutions per site, per year (95% highest posterior density [HPD], 5.33 × 10^–5^–9.994 × 10^–5^).

### Phylogenetic Inference and Phylogeography of SAV in Norwegian Aquaculture

**FIGURE 1:**
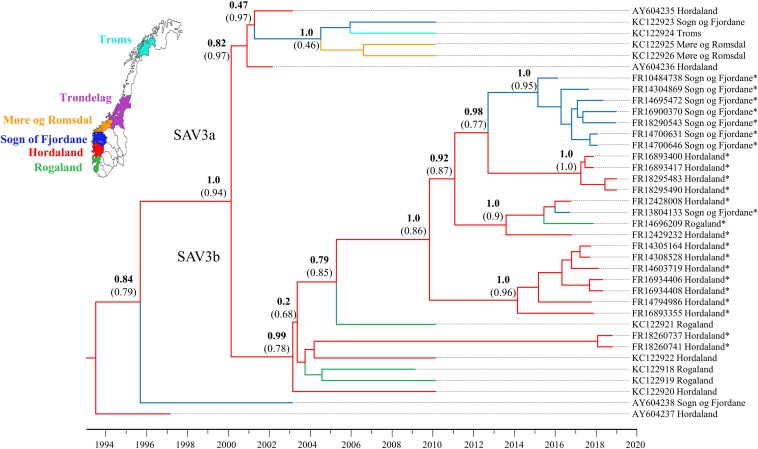
Bayesian phylogeny of the 24 SAV3 genomes generated in this study along with all publicly available SAV3 genome sequences from NCBI. The tree was built from an 11,681 bp alignment and analyzed in BEAST2 using the best fit nucleotide substitution model (TIM2 + G4), a relaxed molecular clock model, tip-dating, and a coalescent Bayesian Skyline population model. A discrete phylogeographical analysis was performed using ancestral reconstruction with branch colors indicating the estimated geographic location of each node. Statistical support for key nodes is indicated by posterior probability values in bold, and the ancestral location probability in brackets. Strains sequenced in this study are indicated by an *.

**FIGURE 2:**
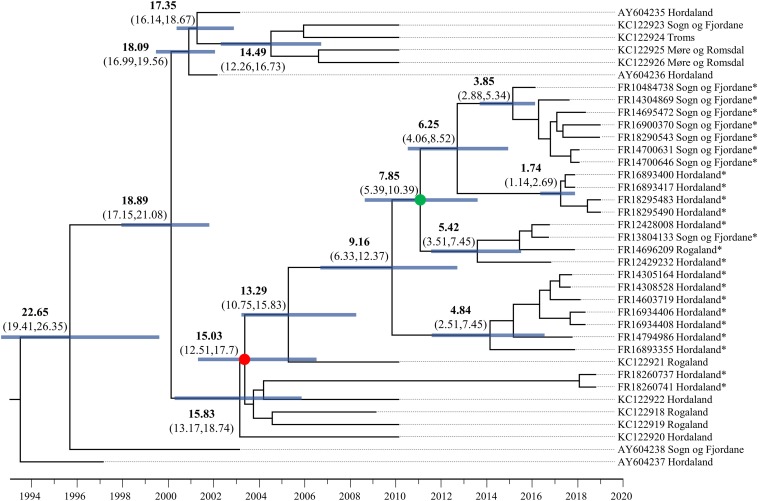
Dated phylogenetic tree of SAV3 built from an 11,681 alignment and analyzed in BEAST2 using the best fit nucleotide substitution model (TIM2 + G4), a relaxed molecular clock model, tip-dating, and a coalescent Bayesian Skyline population model. The values on branches indicate years before 2019 in bold, and the 95% highest posterior density (HPD) values in brackets. Node bars represent 95% HPD age range. Key nodes are indicated with colored circles: the green-colored node is the common ancestor of the Sogn og Fjordane and Rogaland samples in this study, and the red-colored node represents the common ancestor of all samples sequenced in this study. Strains sequenced in this study are indicated by an *.

To better understand patterns of SAV movement in Norwegian aquaculture, we performed a Bayesian phylogeographic analysis (Lemey et al., 2009) including samples generated in the study, along with other publicly available SAV3 genome sequences (Figure 1). The phylogeny suggests that SAV3 consists of two distinct clades (previously observed in Karlsen et al., 2006; Jansen et al., 2010), here defined as SAV3a and SAV3b (Figure 1), which diverged approximately 18.9 years ago or Feb/March of 2000 (95% HPD, 17.15–21.08 years) (Figure 2), relatively early on in the SAV3 epidemic. These two clades differ by silent substitutions in just two locations in the genome, one in nsP2 (703_K_) and the other in E2 (539_S_). The two sequences that branched basal to the two SAV3 clades contain a variant from each clade, indicating that this may have been the ancestral genotype of SAV3 in Norway before splitting into two clades. Moreover, while strains sampled in 2009–2010 fall in both clades, all of the sequences generated in this study (sampled from 2016 to 2019) fall into the SAV3b clade and are highly conserved. Interestingly, the absence of any SAV3a clade strains in the sequences generated in this study – even in samples from the heavily populated Hordaland region - suggests that this lineage may have gone extinct. While the estimated backbone of the phylogeny is Hordaland (Figure 1), there appears to be several distinct lineages that share a MRCA that is 7.85 years (95% HPD, 5.39–10.39 years) or older (Figure 2; green-colored node). The oldest lineage currently active has been evolving separately for around 15 years (95% HPD, 12.51–17.7 years), which indicates that several small-scale, local epidemics are co-circulating at the same time (Figure 2; indicated by red-colored node). All but one of the strains from Sogn of Fjordane were monophyletic, indicating that an individual seeding event from Hordaland (independent of previous strains in this county) resulted in this outbreak. While only a single strain from Rogaland was sequenced in this study, it branched internal to many Hordaland strains, again indicating that the ancestor of this strain originated in Hordaland and was recently introduced to Rogaland.

### Characterization of Structural Deletions in Natural SAV Infections

**FIGURE 3:**
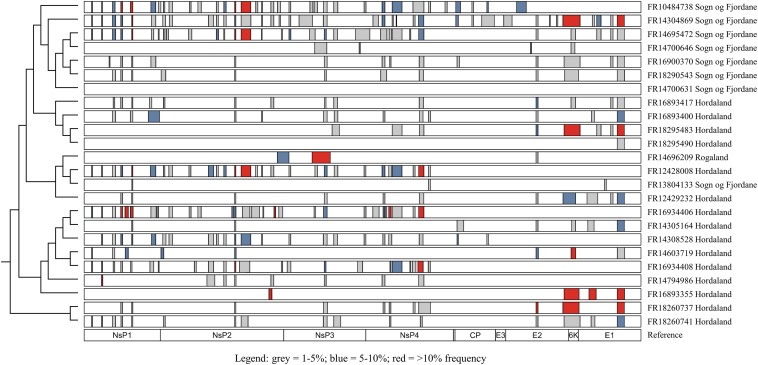
Distribution of deletions (≥10 bp) throughout the SAV3 genome of isolates sequenced on the MinION platform. Only deletions with >50 supporting reads were considered, and all deletions were manually inspected to reduce the rate of false-positive calls. Bars indicate regions with a deletion and are colored by estimated frequency. Isolates are plotted according to phylogenetic relationships shown elsewhere in this study (Figure 1) and the genomic position of each gene is used as a reference.

Natural SAV3 infections have previously been shown to possess numerous defective genomes characterized by deletion variants (Petterson et al., 2013). To further explore this finding, all samples sequenced on the MinION platform were screened for structural variants. The size of deletions detected ranged widely between and within samples, from 11 bp (FR16934408 and FR14304869) to 378 bp (FR14696209). In all but one of the samples (FR14700631) deletions were found, again with a wide range of prevalence (between 1 and 40 deletions per sample). While deletions were found in all genes of the SAV genome, they were not evenly distributed across genes, with relatively fewer deletions detected in the capsid and E3 genes (Figure 3). Several strains from different fish contained the deletions in the same or similar loci (Figure 3). While some closely related strains contained similar deletion variants, which may have been transmitted from one fish to another in the event of a viral genome containing an in-frame deletion being packaged into an infective virion (see “Discussion”), there was little phylogenetic signal in the overall distribution of many apparently similar deletions across the tested samples. Additionally, the estimated frequency of commonly observed deletions varied widely across samples, including closely related strains (Figure 3).

## Discussion

In this study, 24 near-complete genomes were generated from SAV3-positive samples, more than doubling the publicly available genome sequences of Norwegian SAV. We show that SAV3, similar to other alphaviruses (Tan et al., 2018; Ling et al., 2019), evolves relatively slowly in comparison to many RNA viruses with an estimated evolutionary rate of 7.351 × 10^–5^ substitutions per site per year. This substitution rate is similar to previous whole genome estimates but slower than reported for shorter fragments of the genome (e.g., E2) (Karlsen et al., 2014). However, sufficient genetic variation exists within Norwegian SAV3 strains to make genome sequences informative for fine-scale reconstructions of SAV evolution and phylogeographic patterns. Additionally, the temporal signal of SAV3 determined by a TempEst analysis showed strong clock-like signal, and thus further supports the use of genomic sequences in epidemiological studies. The contemporary SAV3 sequences sampled in 2016–2019 were distributed into five distinct clades that were estimated to have had a common ancestor 15 years ago, likely in Hordaland (Figures 1, 2). These five clades were all represented in a relatively limited geographical area of Western Norway during the same timeframe. The clades did not show any clear, long-term geographical pattern within Western Norway, and sequences from Sogn og Fjordane and Rogaland all shared a common ancestor with sequences from Hordaland, which was less than 8 years old, again likely in Hordaland (Figure 2; indicated by the green node). This is much more recent than the first reports of SAV3 in these counties and suggests repeated reintroductions to these areas; our phylogeographic analysis suggested that a Hordaland reservoir was the more likely source. Furthermore, we could not find evidence for long-term (>10 years) persistence of local SAV3 reservoirs in Sogn og Fjordane and Rogaland, suggesting that local epidemics (i.e., outbreaks of related strains that have persisted in a geographic region for longer than any individual cohort of salmon is out to sea) eventually burn out in these areas. While the presence of defective viruses may affect the infection dynamics of individual fish due to strong antiviral responses (see below), the accumulation of defective viral genomes is unlikely to affect epidemic dynamics. A more likely explanation to these apparent local epidemic burn outs is that the density of hosts in “sink” regions (i.e., Rogaland and Sogn og Fjordane) is too low to support long-term SAV3 epidemics. However, it is possible that older SAV3 strains still persist in these sink regions, though no evidence of this was detected in this study.

Considering the presence of sequence diversity within each host, we considered the consensus sequence (i.e., the most abundant viral strain in a sample) to be informative for epidemiological analysis. Our data suggests that Hordaland sources are seemingly seeding introductions of SAV3 strains to surrounding counties where pathogen persistence is shorter than in Hordaland (i.e., Rogaland and Sogn og Fjordane). This dynamic is compatible with a source-sink model (Tan et al., 2018) where viral lineages move from an area which can support a sustained epidemic (the source) to regions that cannot support the epidemic indefinitely (the sink). This is particularly apparent when comparing strains sampled in 2010 and those sampled between 2016 and 2019. In 2010, there were two distinct co-circulating clades of SAV3 (Figure 1). However, samples from 2016 to 2019 fell into only one of these clades, and while this data cannot confirm the extinction of SAV3a, the absence of any recent SAV3a strains supports a source-sink model. These two SAV3 clades have been reported before (Karlsen et al., 2006; Jansen et al., 2010), however, the timing of the split between the two clades had not yet been estimated. It is somewhat unsurprising to find Hordaland as an occasional source of virus, since the county has the highest density of seawater sites and the highest number of reported SAV3 cases per year (Norwegian Veterinary Institute, 2018). The lack of long-term established SAV local reservoirs in Sogn og Fjordane and Rogaland is, however, more noteworthy. A possible explanation to this pattern could be that a significant number of SAV3 transmissions across large geographic distances are not a result of passive transport with water currents, but rather of anthropogenic origin. However, it is still likely that passive transport plays a role in short-term local outbreaks.

An interesting finding was the presence of multiple presumably defective viral RNA sequences carrying numerous deletions across the genome (Figure 3). All samples apart from one contained deletions ranging from 1 to 40 deletions per isolate, across a range of lengths from 11 bp to 378 bp. Deletion mutations have previously been described in Venezuelan equine encephalitis, a closely related member of the alphavirus genus (Forrester et al., 2011), and in SAV (Petterson et al., 2013, 2016), with the latter showing the presence of the same or similar deletions in multiple samples. The non-random distribution of deletions across the SAV genome, seen clearly in our analysis (Figure 3), suggests that template switching during RNA synthesis is a plausible source of origin, with factors such as secondary RNA structure, sequence identity and the kinetics of transcription influencing template switching (Baird et al., 2006; Simon-Loriere and Holmes, 2011). While it is unlikely that viral genomes missing large sections of either the structural or non-structural polyprotein are viable, it is impossible to rule that all of the deletions observed result in a defective viral particle, considering that approximately 34% of the deletions did not cause a disruption to the protein coding sequence (in-frame deletions) (Supplementary Table S1). Additionally, in-frame deletions located in the non-structural polyprotein may only affect the replication of the virus, not the packaging of the virus particles, though more research into this area would shed light on the impact of such deletions on the packaging signals of SAV. As SAV has been shown to recombine *in vivo* to rescue viral genomes with otherwise fatal mutation errors (Petterson et al., 2016), it is possible that defective RNA molecules may be packaged into viral particles in the eventuality that more than one particle infects a single cell. Additionally, very similar deletions were found in closely related strains (Figure 3), which is compatible with deleted genomes being transmitted between infected fish. However, equally similar deletions are found across relatively distant strains which strongly suggests an independent origin of at least the majority of the deletion mutations.

Finally, as several of the samples used in this study were from regions that have had outbreaks of both SAV2 and SAV3 in the past, our sequencing method was tested on the sensitivity and accuracy of detecting both subtypes in the same sample. Our previous study has shown that multiple SAV subtypes are commonly co-circulating on the same farm, and may also be found as co-infections within individual fish (Gallagher et al., 2020). At an error rate of less than 0.3%, our results show that sequencing ∼2 kb amplicons and sequencing on the MinION platform enables highly accurate detection of such co-infections, even when the second subtype is at a relatively low titer (as low as 5% of the total SAV reads). However, these data are based on *in silico* co-infections as none of the samples sequenced in this study showed natural co-infections of SAV2 and SAV3, and future work should include samples that have been mixed in the lab prior to sequencing, as well as natural co-infections when identified.

In conclusion, whole genome analyses has helped increase our knowledge of the genetic diversity found in SAV infections impacting farmed salmon, and can thus be used to understand transmission pathways, viral population dynamics and the potential role that wild reservoirs play in ongoing PD epidemics. The apparent repeated seeding of SAV3 from “source” counties like Hordaland to surrounding “sink” counties implies that effective mitigating strategies might be able to limit the PD epidemic in “sink” regions with improved biosecurity approaches. Future investigations into this would benefit from higher geographical resolution than was available in this study (i.e., at the farm level). However, more work is required to understand the relative impact that passive transmission (i.e., water currents) has on viral spread compared to the transportation of viral material via infected fish, especially on different geographical and temporal scales.

## Data Availability Statement

Raw sequence files are available under SRA BioProject PRJNA599578. Genome sequences are available in Genbank under the accession numbers: MN906915–MN906938.

## Ethics Statement

No animal experiments were performed during this study. Viral strains were isolated from fish specimens derived from the diagnostic and monitoring activities carried out at PHARMAQ Analytik, Bergen, Norway. Surveillance, both active and passive, was performed by competent local veterinary authorities according to the relevant legislation (Directive 91/67/EC; Directive 2006/88/EC) through the periodical inspections of farms as well as samples collection in case of clinical suspicion.

## Author Contributions

All authors devised the study and contributed to writing, improving the manuscript draft, and approved the final submitted manuscript. MG performed lab-work and analysis, drafted the manuscript, figures and tables. MK, EP, and ØH provided samples.

## Conflict of Interest

MK, EP, and ØH are employed by PHARMAQ AS. The remaining authors declare that the research was conducted in the absence of any commercial or financial relationships that could be construed as a potential conflict of interest.

## Footnotes

1 http://tree.bio.ed.ac.uk/software/figtree/
