# Supplementary material for: Genome Sequencing of SAV3 Reveals Repeated Seeding Events of Viral Strains in Norwegian Aquaculture
Source: Front Microbiol. 2020 Apr 24;11:740. doi: 10.3389/fmicb.2020.00740 (PMC7193772; doi:10.3389/fmicb.2020.00740)
Supplement: Supplementary file 1 [file Table_1.DOCX]

**Table S1.** Summary of deletions characterised in 24 naturally infected SAV3 samples from Norway.

| **Sample_ID** | **start** | **end** | **Length** | **Effect** | **Gene** |
| --- | --- | --- | --- | --- | --- |
| SAV3_BC01 | 363 | 383 | 20 | Frameshift | nsP1 |
| SAV3_BC01 | 592 | 657 | 65 | Frameshift | nsP1 |
| SAV3_BC01 | 768 | 803 | 35 | Frameshift | nsP1 |
| SAV3_BC01 | 996 | 1018 | 22 | Frameshift | nsP1 |
| SAV3_BC01 | 1409 | 1498 | 89 | Frameshift | nsP1 |
| SAV3_BC01 | 1644 | 1709 | 65 | Frameshift | nsP2 |
| SAV3_BC01 | 1773 | 1844 | 71 | Frameshift | nsP2 |
| SAV3_BC01 | 2599 | 2706 | 107 | Frameshift | nsP2 |
| SAV3_BC01 | 2764 | 2804 | 40 | Frameshift | nsP2 |
| SAV3_BC01 | 2919 | 2970 | 51 | In-frame | nsP2 |
| SAV3_BC01 | 3147 | 3171 | 24 | In-frame | nsP2 |
| SAV3_BC01 | 3281 | 3482 | 201 | In-frame | nsP2 |
| SAV3_BC01 | 3707 | 3726 | 19 | Frameshift | nsP2 |
| SAV3_BC01 | 4282 | 4318 | 36 | In-frame | nsP3 |
| SAV3_BC01 | 4999 | 5079 | 80 | Frameshift | nsP3 |
| SAV3_BC01 | 5217 | 5296 | 79 | Frameshift | nsP3 |
| SAV3_BC01 | 5850 | 5888 | 38 | Frameshift | nsP3-nsP4 |
| SAV3_BC01 | 6244 | 6286 | 42 | In-frame | nsP4 |
| SAV3_BC01 | 6366 | 6429 | 63 | In-frame | nsP4 |
| SAV3_BC01 | 7002 | 7083 | 81 | In-frame | nsP4 |
| SAV3_BC01 | 7793 | 7821 | 28 | Frameshift | Cp |
| SAV3_BC01 | 8417 | 8447 | 30 | In-frame | Cp |
| SAV3_BC02 | 997 | 1019 | 22 | Frameshift | nsP1 |
| SAV3_BC02 | 3147 | 3171 | 24 | In-frame | nsP2 |
| SAV3_BC02 | 7793 | 7931 | 138 | In-frame | Cp |
| SAV3_BC02 | 9443 | 9484 | 41 | Frameshift | E2 |
| SAV3_BC02 | 10190 | 10269 | 79 | Frameshift | 6K |
| SAV3_BC02 | 10526 | 10656 | 130 | Frameshift | E1 |
| SAV3_BC02 | 11140 | 11288 | 148 | Frameshift | E1 |
| SAV3_BC03 | 594 | 657 | 63 | In-frame | nsP1 |
| SAV3_BC03 | 969 | 1018 | 49 | Frameshift | nsP1 |
| SAV3_BC03 | 1342 | 1574 | 232 | Frameshift | nsP1 |
| SAV3_BC03 | 3147 | 3172 | 25 | Frameshift | nsP2 |
| SAV3_BC03 | 3707 | 3727 | 20 | Frameshift | nsP2 |
| SAV3_BC03 | 5000 | 5079 | 79 | Frameshift | nsP3 |
| SAV3_BC03 | 5227 | 5302 | 75 | In-frame | nsP3 |
| SAV3_BC03 | 6246 | 6286 | 40 | Frameshift | nsP4 |
| SAV3_BC03 | 7010 | 7083 | 73 | Frameshift | nsP4 |
| SAV3_BC03 | 7196 | 7239 | 43 | Frameshift | nsP4 |
| SAV3_BC03 | 9443 | 9485 | 42 | In-frame | E2 |
| SAV3_BC03 | 10605 | 10661 | 56 | Frameshift | E1 |
| SAV3_BC03 | 11140 | 11289 | 149 | Frameshift | E1 |
| SAV3_BC04 | 608 | 639 | 31 | Frameshift | nsP1 |
| SAV3_BC04 | 767 | 803 | 36 | In-frame | nsP1 |
| SAV3_BC04 | 998 | 1018 | 20 | Frameshift | nsP1 |
| SAV3_BC04 | 1367 | 1414 | 47 | Frameshift | nsP1 |
| SAV3_BC04 | 1660 | 1688 | 28 | Frameshift | nsP2 |
| SAV3_BC04 | 3147 | 3172 | 25 | Frameshift | nsP2 |
| SAV3_BC04 | 3904 | 3964 | 60 | In-frame | nsP2 |
| SAV3_BC04 | 5024 | 5055 | 31 | Frameshift | nsP3 |
| SAV3_BC04 | 5217 | 5296 | 79 | Frameshift | nsP3 |
| SAV3_BC04 | 7002 | 7089 | 87 | In-frame | nsP4 |
| SAV3_BC04 | 9443 | 9485 | 42 | In-frame | E2 |
| SAV3_BC04 | 10175 | 10269 | 94 | Frameshift | 6K |
| SAV3_BC04 | 11006 | 11052 | 46 | Frameshift | E1 |
| SAV3_BC04 | 11140 | 11288 | 148 | Frameshift | E1 |
| SAV3_BC05 | 3863 | 3923 | 60 | In-frame | nsP2 |
| SAV3_BC05 | 10028 | 10343 | 315 | In-frame | E2-6K |
| SAV3_BC05 | 10551 | 10700 | 149 | Frameshift | E1 |
| SAV3_BC05 | 11140 | 11291 | 151 | Frameshift | E1 |
| SAV3_BC07 | 4820 | 5070 | 250 | Frameshift | nsP3 |
| SAV3_BC07 | 5749 | 5771 | 22 | Frameshift | nsP3 |
| SAV3_BC07 | 9329 | 9347 | 18 | In-frame | E2 |
| SAV3_BC07 | 10175 | 10256 | 81 | In-frame | 6K |
| SAV3_BC09 | 157 | 176 | 19 | Frameshift | nsP1 |
| SAV3_BC09 | 593 | 640 | 47 | Frameshift | nsP1 |
| SAV3_BC09 | 859 | 921 | 62 | Frameshift | nsP1 |
| SAV3_BC09 | 1606 | 1664 | 58 | Frameshift | nsP2 |
| SAV3_BC09 | 3147 | 3176 | 29 | Frameshift | nsP2 |
| SAV3_BC09 | 9443 | 9490 | 47 | Frameshift | E2 |
| SAV3_BC09 | 10176 | 10273 | 97 | Frameshift | 6K |
| SAV3_BC09 | 11143 | 11293 | 150 | In-frame | E1 |
| SAV3_BC11 | 157 | 176 | 19 | Frameshift | nsP1 |
| SAV3_BC11 | 366 | 384 | 18 | In-frame | nsP1 |
| SAV3_BC11 | 592 | 657 | 65 | Frameshift | nsP1 |
| SAV3_BC11 | 768 | 802 | 34 | Frameshift | nsP1 |
| SAV3_BC11 | 997 | 1018 | 21 | In-frame | nsP1 |
| SAV3_BC11 | 1409 | 1454 | 45 | In-frame | nsP1 |
| SAV3_BC11 | 1549 | 1567 | 18 | In-frame | nsP1 |
| SAV3_BC11 | 1660 | 1689 | 29 | Frameshift | nsP2 |
| SAV3_BC11 | 1719 | 1761 | 42 | In-frame | nsP2 |
| SAV3_BC11 | 2185 | 2250 | 65 | Frameshift | nsP2 |
| SAV3_BC11 | 2764 | 2806 | 42 | In-frame | nsP2 |
| SAV3_BC11 | 2919 | 2938 | 19 | Frameshift | nsP2 |
| SAV3_BC11 | 3147 | 3171 | 24 | In-frame | nsP2 |
| SAV3_BC11 | 3281 | 3482 | 201 | In-frame | nsP2 |
| SAV3_BC11 | 3707 | 3720 | 13 | Frameshift | nsP2 |
| SAV3_BC11 | 4120 | 4144 | 24 | In-frame | nsP2 |
| SAV3_BC11 | 4282 | 4320 | 38 | Frameshift | nsP3 |
| SAV3_BC11 | 4710 | 4827 | 117 | In-frame | nsP3 |
| SAV3_BC11 | 5020 | 5060 | 40 | Frameshift | nsP3 |
| SAV3_BC11 | 5217 | 5296 | 79 | Frameshift | nsP3 |
| SAV3_BC11 | 5674 | 5972 | 298 | Frameshift | nsP3 |
| SAV3_BC11 | 6197 | 6327 | 130 | Frameshift | nsP4 |
| SAV3_BC11 | 6374 | 6418 | 44 | Frameshift | nsP4 |
| SAV3_BC11 | 6438 | 6644 | 206 | Frameshift | nsP4 |
| SAV3_BC11 | 6805 | 6825 | 20 | Frameshift | nsP4 |
| SAV3_BC11 | 6993 | 7101 | 108 | In-frame | nsP4 |
| SAV3_BC11 | 9443 | 9485 | 42 | In-frame | E2 |
| SAV3_BC11 | 10175 | 10269 | 94 | Frameshift | 6K |
| SAV3_BC11 | 10713 | 10801 | 88 | Frameshift | E1 |
| SAV3_BC11 | 11006 | 11052 | 46 | Frameshift | E1 |
| SAV3_BC11 | 11140 | 11289 | 149 | Frameshift | E1 |
| SAV3_BC12 | 158 | 178 | 20 | Frameshift | nsP1 |
| SAV3_BC12 | 366 | 383 | 17 | Frameshift | nsP1 |
| SAV3_BC12 | 608 | 639 | 31 | Frameshift | nsP1 |
| SAV3_BC12 | 768 | 802 | 34 | Frameshift | nsP1 |
| SAV3_BC12 | 970 | 999 | 29 | Frameshift | nsP1 |
| SAV3_BC12 | 1376 | 1455 | 79 | Frameshift | nsP1 |
| SAV3_BC12 | 1549 | 1566 | 17 | Frameshift | nsP1 |
| SAV3_BC12 | 2304 | 2343 | 39 | In-frame | nsP2 |
| SAV3_BC12 | 2599 | 2705 | 106 | Frameshift | nsP2 |
| SAV3_BC12 | 2919 | 2970 | 51 | In-frame | nsP2 |
| SAV3_BC12 | 3147 | 3171 | 24 | In-frame | nsP2 |
| SAV3_BC12 | 3248 | 3462 | 214 | Frameshift | nsP2 |
| SAV3_BC12 | 4120 | 4146 | 26 | Frameshift | nsP2 |
| SAV3_BC12 | 4243 | 4320 | 77 | Frameshift | nsP3 |
| SAV3_BC12 | 5020 | 5055 | 35 | Frameshift | nsP3 |
| SAV3_BC12 | 5716 | 5819 | 103 | Frameshift | nsP3 |
| SAV3_BC12 | 6392 | 6410 | 18 | In-frame | nsP4 |
| SAV3_BC12 | 6438 | 6642 | 204 | In-frame | nsP4 |
| SAV3_BC12 | 6807 | 6826 | 19 | Frameshift | nsP4 |
| SAV3_BC12 | 6861 | 6933 | 72 | In-frame | nsP4 |
| SAV3_BC12 | 6975 | 7084 | 109 | Frameshift | nsP4 |
| SAV3_BC12 | 7191 | 7233 | 42 | In-frame | nsP4 |
| SAV3_BC13 | 157 | 177 | 20 | Frameshift | nsP1 |
| SAV3_BC13 | 367 | 384 | 17 | Frameshift | nsP1 |
| SAV3_BC13 | 594 | 657 | 63 | In-frame | nsP1 |
| SAV3_BC13 | 765 | 802 | 37 | Frameshift | nsP1 |
| SAV3_BC13 | 855 | 921 | 66 | In-frame | nsP1 |
| SAV3_BC13 | 968 | 1022 | 54 | In-frame | nsP1 |
| SAV3_BC13 | 1395 | 1506 | 111 | In-frame | nsP1 |
| SAV3_BC13 | 1524 | 1553 | 29 | Frameshift | nsP1 |
| SAV3_BC13 | 1773 | 1845 | 72 | In-frame | nsP2 |
| SAV3_BC13 | 2194 | 2233 | 39 | In-frame | nsP2 |
| SAV3_BC13 | 2376 | 2445 | 69 | In-frame | nsP2 |
| SAV3_BC13 | 3086 | 3120 | 34 | Frameshift | nsP2 |
| SAV3_BC13 | 3147 | 3172 | 25 | Frameshift | nsP2 |
| SAV3_BC13 | 3308 | 3463 | 155 | Frameshift | nsP2 |
| SAV3_BC13 | 3502 | 3665 | 163 | Frameshift | nsP2 |
| SAV3_BC13 | 3737 | 3755 | 18 | In-frame | nsP2 |
| SAV3_BC13 | 3969 | 4001 | 32 | Frameshift | nsP2 |
| SAV3_BC13 | 4069 | 4150 | 81 | In-frame | nsP2 |
| SAV3_BC13 | 4805 | 4831 | 26 | Frameshift | nsP3 |
| SAV3_BC13 | 5000 | 5079 | 79 | Frameshift | nsP3 |
| SAV3_BC13 | 5850 | 5890 | 40 | Frameshift | nsP3-nsP4 |
| SAV3_BC13 | 6033 | 6163 | 130 | Frameshift | nsP4 |
| SAV3_BC13 | 6246 | 6282 | 36 | In-frame | nsP4 |
| SAV3_BC13 | 6301 | 6323 | 22 | Frameshift | nsP4 |
| SAV3_BC13 | 6366 | 6413 | 47 | Frameshift | nsP4 |
| SAV3_BC13 | 6436 | 6642 | 206 | Frameshift | nsP4 |
| SAV3_BC13 | 6875 | 6915 | 40 | Frameshift | nsP4 |
| SAV3_BC13 | 6993 | 7101 | 108 | In-frame | nsP4 |
| SAV3_BC14 | 157 | 177 | 20 | Frameshift | nsP1 |
| SAV3_BC14 | 363 | 383 | 20 | Frameshift | nsP1 |
| SAV3_BC14 | 594 | 656 | 62 | Frameshift | nsP1 |
| SAV3_BC14 | 772 | 803 | 31 | Frameshift | nsP1 |
| SAV3_BC14 | 996 | 1018 | 22 | Frameshift | nsP1 |
| SAV3_BC14 | 1656 | 1694 | 38 | Frameshift | nsP2 |
| SAV3_BC14 | 3147 | 3171 | 24 | In-frame | nsP2 |
| SAV3_BC14 | 3281 | 3426 | 145 | Frameshift | nsP2 |
| SAV3_BC14 | 4999 | 5079 | 80 | Frameshift | nsP3 |
| SAV3_BC14 | 5217 | 5361 | 144 | In-frame | nsP3 |
| SAV3_BC14 | 6385 | 6410 | 25 | Frameshift | nsP4 |
| SAV3_BC14 | 7025 | 7065 | 40 | Frameshift | nsP4 |
| SAV3_BC14 | 9443 | 9484 | 41 | Frameshift | E2 |
| SAV3_BC14 | 10028 | 10364 | 336 | In-frame | E2-6K |
| SAV3_BC14 | 10594 | 10674 | 80 | Frameshift | E1 |
| SAV3_BC14 | 11140 | 11288 | 148 | Frameshift | E1 |
| SAV3_BC15 | 768 | 802 | 34 | Frameshift | nsP1 |
| SAV3_BC15 | 997 | 1019 | 22 | Frameshift | nsP1 |
| SAV3_BC15 | 3147 | 3171 | 24 | In-frame | nsP2 |
| SAV3_BC15 | 5002 | 5082 | 80 | Frameshift | nsP3 |
| SAV3_BC15 | 6246 | 6286 | 40 | Frameshift | nsP4 |
| SAV3_BC15 | 6375 | 6410 | 35 | Frameshift | nsP4 |
| SAV3_BC15 | 6875 | 6915 | 40 | Frameshift | nsP4 |
| SAV3_BC15 | 6994 | 7236 | 242 | Frameshift | nsP4 |
| SAV3_BC15 | 9443 | 9485 | 42 | In-frame | E2 |
| SAV3_BC15 | 10004 | 10343 | 339 | In-frame | E2-6K |
| SAV3_BC15 | 11140 | 11289 | 149 | Frameshift | E1 |
| SAV3_BC16 | 592 | 657 | 65 | Frameshift | nsP1 |
| SAV3_BC16 | 768 | 802 | 34 | Frameshift | nsP1 |
| SAV3_BC16 | 996 | 1018 | 22 | Frameshift | nsP1 |
| SAV3_BC16 | 1611 | 1706 | 95 | Frameshift | nsP2 |
| SAV3_BC16 | 3147 | 3171 | 24 | In-frame | nsP2 |
| SAV3_BC16 | 5024 | 5058 | 34 | Frameshift | nsP3 |
| SAV3_BC16 | 6198 | 6322 | 124 | Frameshift | nsP4 |
| SAV3_BC16 | 7002 | 7089 | 87 | In-frame | nsP4 |
| SAV3_BC16 | 9444 | 9487 | 43 | Frameshift | E2 |
| SAV3_BC16 | 10043 | 10331 | 288 | In-frame | E2-6K |
| SAV3_BC16 | 11141 | 11291 | 150 | In-frame | E1 |
| SAV3_BC17 | 521 | 541 | 20 | Frameshift | nsP1 |
| SAV3_BC17 | 768 | 803 | 35 | Frameshift | nsP1 |
| SAV3_BC17 | 997 | 1018 | 21 | In-frame | nsP1 |
| SAV3_BC17 | 1395 | 1499 | 104 | Frameshift | nsP1 |
| SAV3_BC17 | 3147 | 3171 | 24 | In-frame | nsP2 |
| SAV3_BC17 | 5020 | 5079 | 59 | Frameshift | nsP3 |
| SAV3_BC17 | 6227 | 6286 | 59 | Frameshift | nsP4 |
| SAV3_BC17 | 6387 | 6412 | 25 | Frameshift | nsP4 |
| SAV3_BC17 | 7002 | 7101 | 99 | In-frame | nsP4 |
| SAV3_BC17 | 7793 | 7844 | 51 | In-frame | Cp |
| SAV3_BC17 | 9443 | 9484 | 41 | Frameshift | E2 |
| SAV3_BC17 | 10028 | 10364 | 336 | In-frame | E2-6K |
| SAV3_BC17 | 11006 | 11052 | 46 | Frameshift | E1 |
| SAV3_BC17 | 11140 | 11289 | 149 | Frameshift | E1 |
| SAV3_BC18 | 11140 | 11289 | 149 | Frameshift | E1 |
| SAV3_BC20 | 365 | 389 | 24 | In-frame | nsP1 |
| SAV3_BC20 | 2567 | 2730 | 163 | Frameshift | nsP2 |
| SAV3_BC20 | 2919 | 2970 | 51 | In-frame | nsP2 |
| SAV3_BC20 | 3147 | 3173 | 26 | Frameshift | nsP2 |
| SAV3_BC20 | 3904 | 3963 | 59 | Frameshift | nsP2 |
| SAV3_BC20 | 5020 | 5057 | 37 | Frameshift | nsP3 |
| SAV3_BC20 | 6227 | 6286 | 59 | Frameshift | nsP4 |
| SAV3_BC20 | 6993 | 7101 | 108 | In-frame | nsP4 |
| SAV3_BC21 | 5182 | 5332 | 150 | In-frame | nsP3 |
| SAV3_BC21 | 6438 | 6642 | 204 | In-frame | nsP4 |
| SAV3_BC21 | 7004 | 7101 | 97 | Frameshift | nsP4 |
| SAV3_BC21 | 9443 | 9484 | 41 | Frameshift | E2 |
| SAV3_BC21 | 10028 | 10364 | 336 | In-frame | E2-6K |
| SAV3_BC21 | 10713 | 10801 | 88 | Frameshift | E1 |
| SAV3_BC21 | 11006 | 11052 | 46 | Frameshift | E1 |
| SAV3_BC21 | 11140 | 11289 | 149 | Frameshift | E1 |
| SAV3_BC22 | 4040 | 4281 | 241 | Frameshift | nsP2 |
| SAV3_BC22 | 4764 | 5142 | 378 | In-frame | nsP3 |
| SAV3_BC22 | 9443 | 9484 | 41 | Frameshift | E2 |
| SAV3_BC23 | 768 | 803 | 35 | Frameshift | nsP1 |
| SAV3_BC23 | 996 | 1018 | 22 | Frameshift | nsP1 |
| SAV3_BC23 | 3147 | 3171 | 24 | In-frame | nsP2 |
| SAV3_BC23 | 5020 | 5079 | 59 | Frameshift | nsP3 |
| SAV3_BC23 | 6246 | 6286 | 40 | Frameshift | nsP4 |
| SAV3_BC23 | 7004 | 7099 | 95 | Frameshift | nsP4 |
| SAV3_BC23 | 9443 | 9484 | 41 | Frameshift | E2 |
| SAV3_BC23 | 10007 | 10269 | 262 | Frameshift | E2-6K |
| SAV3_BC23 | 10509 | 10733 | 224 | Frameshift | E1 |
| SAV3_BC23 | 11006 | 11052 | 46 | Frameshift | E1 |
| SAV3_BC23 | 11140 | 11289 | 149 | Frameshift | E1 |
| SAV3_BC24 | 157 | 176 | 19 | Frameshift | nsP1 |
| SAV3_BC24 | 363 | 384 | 21 | In-frame | nsP1 |
| SAV3_BC24 | 592 | 657 | 65 | Frameshift | nsP1 |
| SAV3_BC24 | 768 | 803 | 35 | Frameshift | nsP1 |
| SAV3_BC24 | 997 | 1018 | 21 | In-frame | nsP1 |
| SAV3_BC24 | 1387 | 1498 | 111 | In-frame | nsP1 |
| SAV3_BC24 | 1660 | 1686 | 26 | Frameshift | nsP2 |
| SAV3_BC24 | 1771 | 1844 | 73 | Frameshift | nsP2 |
| SAV3_BC24 | 2191 | 2232 | 41 | Frameshift | nsP2 |
| SAV3_BC24 | 2599 | 2706 | 107 | Frameshift | nsP2 |
| SAV3_BC24 | 2764 | 2804 | 40 | Frameshift | nsP2 |
| SAV3_BC24 | 2919 | 2970 | 51 | In-frame | nsP2 |
| SAV3_BC24 | 3147 | 3171 | 24 | In-frame | nsP2 |
| SAV3_BC24 | 3281 | 3482 | 201 | In-frame | nsP2 |
| SAV3_BC24 | 3582 | 3625 | 43 | Frameshift | nsP2 |
| SAV3_BC24 | 3707 | 3727 | 20 | Frameshift | nsP2 |
| SAV3_BC24 | 3904 | 3964 | 60 | In-frame | nsP2 |
| SAV3_BC24 | 4282 | 4318 | 36 | In-frame | nsP3 |
| SAV3_BC24 | 4825 | 4873 | 48 | In-frame | nsP3 |
| SAV3_BC24 | 5020 | 5079 | 59 | Frameshift | nsP3 |
| SAV3_BC24 | 5225 | 5301 | 76 | Frameshift | nsP3 |
| SAV3_BC24 | 5850 | 5888 | 38 | Frameshift | nsP3-nsP4 |
| SAV3_BC24 | 6227 | 6286 | 59 | Frameshift | nsP4 |
| SAV3_BC24 | 6366 | 6429 | 63 | In-frame | nsP4 |
| SAV3_BC24 | 6438 | 6641 | 203 | Frameshift | nsP4 |
| SAV3_BC24 | 6876 | 6914 | 38 | Frameshift | nsP4 |
| SAV3_BC24 | 6993 | 7101 | 108 | In-frame | nsP4 |
| SAV3_BC24 | 7197 | 7235 | 38 | Frameshift | nsP4 |
| SAV3_BC25 | 998 | 1018 | 20 | Frameshift | nsP1 |
| SAV3_BC25 | 7197 | 7236 | 39 | In-frame | nsP4 |
| SAV3_BC25 | 10876 | 10911 | 35 | Frameshift | E1 |
| SAV3_BC26 | 157 | 176 | 19 | Frameshift | nsP1 |
| SAV3_BC26 | 363 | 383 | 20 | Frameshift | nsP1 |
| SAV3_BC26 | 592 | 657 | 65 | Frameshift | nsP1 |
| SAV3_BC26 | 768 | 803 | 35 | Frameshift | nsP1 |
| SAV3_BC26 | 997 | 1018 | 21 | In-frame | nsP1 |
| SAV3_BC26 | 1409 | 1498 | 89 | Frameshift | nsP1 |
| SAV3_BC26 | 1644 | 1701 | 57 | In-frame | nsP2 |
| SAV3_BC26 | 1781 | 1844 | 63 | In-frame | nsP2 |
| SAV3_BC26 | 2599 | 2707 | 108 | In-frame | nsP2 |
| SAV3_BC26 | 2764 | 2804 | 40 | Frameshift | nsP2 |
| SAV3_BC26 | 2919 | 2970 | 51 | In-frame | nsP2 |
| SAV3_BC26 | 3147 | 3171 | 24 | In-frame | nsP2 |
| SAV3_BC26 | 3281 | 3485 | 204 | In-frame | nsP2 |
| SAV3_BC26 | 3707 | 3727 | 20 | Frameshift | nsP2 |
| SAV3_BC26 | 3904 | 3964 | 60 | In-frame | nsP2 |
| SAV3_BC26 | 4073 | 4099 | 26 | Frameshift | nsP2 |
| SAV3_BC26 | 4282 | 4318 | 36 | In-frame | nsP3 |
| SAV3_BC26 | 4491 | 4769 | 278 | Frameshift | nsP3 |
| SAV3_BC26 | 4999 | 5079 | 80 | Frameshift | nsP3 |
| SAV3_BC26 | 6037 | 6154 | 117 | In-frame | nsP4 |
| SAV3_BC26 | 6227 | 6286 | 59 | Frameshift | nsP4 |
| SAV3_BC26 | 6436 | 6459 | 23 | Frameshift | nsP4 |
| SAV3_BC26 | 6521 | 6532 | 11 | Frameshift | nsP4 |
| SAV3_BC26 | 6876 | 6914 | 38 | Frameshift | nsP4 |
| SAV3_BC26 | 6989 | 7099 | 110 | Frameshift | nsP4 |
| SAV3_BC26 | 7766 | 7821 | 55 | Frameshift | Cp |
| SAV3_BC26 | 8104 | 8138 | 34 | Frameshift | Cp |
| SAV3_BC26 | 8309 | 8571 | 262 | Frameshift | Cp |
| SAV3_BC26 | 8769 | 8943 | 174 | In-frame | E3-E2 |
| SAV3_BC26 | 9443 | 9485 | 42 | In-frame | E2 |
| SAV3_BC26 | 9726 | 9741 | 15 | In-frame | E2 |
| SAV3_BC26 | 9873 | 9900 | 27 | In-frame | E2 |
| SAV3_BC26 | 10004 | 10364 | 360 | In-frame | E2-6K |
| SAV3_BC26 | 10620 | 10661 | 41 | Frameshift | E1 |
| SAV3_BC26 | 10713 | 10801 | 88 | Frameshift | E1 |
| SAV3_BC26 | 11006 | 11052 | 46 | Frameshift | E1 |
| SAV3_BC26 | 11140 | 11288 | 148 | Frameshift | E1 |
| SAV3_BC27 | 158 | 176 | 18 | In-frame | nsP1 |
| SAV3_BC27 | 363 | 384 | 21 | In-frame | nsP1 |
| SAV3_BC27 | 592 | 648 | 56 | Frameshift | nsP1 |
| SAV3_BC27 | 768 | 802 | 34 | Frameshift | nsP1 |
| SAV3_BC27 | 969 | 1018 | 49 | Frameshift | nsP1 |
| SAV3_BC27 | 1395 | 1498 | 103 | Frameshift | nsP1 |
| SAV3_BC27 | 1550 | 1603 | 53 | Frameshift | nsP1-nsP2 |
| SAV3_BC27 | 1660 | 1686 | 26 | Frameshift | nsP2 |
| SAV3_BC27 | 1773 | 1844 | 71 | Frameshift | nsP2 |
| SAV3_BC27 | 2203 | 2233 | 30 | In-frame | nsP2 |
| SAV3_BC27 | 2376 | 2446 | 70 | Frameshift | nsP2 |
| SAV3_BC27 | 2764 | 2804 | 40 | Frameshift | nsP2 |
| SAV3_BC27 | 2919 | 2970 | 51 | In-frame | nsP2 |
| SAV3_BC27 | 3147 | 3171 | 24 | In-frame | nsP2 |
| SAV3_BC27 | 3281 | 3482 | 201 | In-frame | nsP2 |
| SAV3_BC27 | 3586 | 3631 | 45 | In-frame | nsP2 |
| SAV3_BC27 | 3707 | 3727 | 20 | Frameshift | nsP2 |
| SAV3_BC27 | 3904 | 3964 | 60 | In-frame | nsP2 |
| SAV3_BC27 | 4040 | 4101 | 61 | Frameshift | nsP2 |
| SAV3_BC27 | 4282 | 4318 | 36 | In-frame | nsP3 |
| SAV3_BC27 | 4720 | 4780 | 60 | In-frame | nsP3 |
| SAV3_BC27 | 4807 | 4874 | 67 | Frameshift | nsP3 |
| SAV3_BC27 | 5001 | 5079 | 78 | In-frame | nsP3 |
| SAV3_BC27 | 5217 | 5301 | 84 | In-frame | nsP3 |
| SAV3_BC27 | 5850 | 5890 | 40 | Frameshift | nsP3-nsP4 |
| SAV3_BC27 | 6227 | 6286 | 59 | Frameshift | nsP4 |
| SAV3_BC27 | 6438 | 6643 | 205 | Frameshift | nsP4 |
| SAV3_BC27 | 6872 | 7101 | 229 | Frameshift | nsP4 |
| SAV3_BC27 | 7200 | 7237 | 37 | Frameshift | nsP4 |
| SAV3_BC27 | 7542 | 7586 | 44 | Frameshift | nsP4 |
| SAV3_BC27 | 7766 | 7865 | 99 | In-frame | Cp |
| SAV3_BC27 | 8104 | 8138 | 34 | Frameshift | Cp |
| SAV3_BC27 | 8417 | 8446 | 29 | Frameshift | Cp |
| SAV3_BC27 | 9037 | 9240 | 203 | Frameshift | E2 |
